# SOCIAL SANDWICHING AND PAID WORK IN LATER LIFE: CONSEQUENCES ON MENTAL HEALTH

**DOI:** 10.1093/geroni/igad104.3596

**Published:** 2023-12-21

**Authors:** Marco Albertini, Noah Lewin-Epstein, Merril Silverstein, Aviad Tur-Sinai

**Affiliations:** Alma Mater Studiorum - Università di Bologna, Bologna, Emilia-Romagna, Italy; Tel Aviv University, Tel Aviv-Yafo, Tel Aviv, Israel; Syracuse University, Syracuse, New York, United States; The Max Stern Yezreel Valley College, Yezreel Valley, HaZafon, Israel

## Abstract

Conflicting demands over time allocation – between family care and paid work – can be harmful to individual’s wellbeing. Individuals who are socially sandwiched and in employment experience potential conflict between supporting older and younger family generations, and with respect to the demands from paid work. These competing time demands may have negative effects on individuals’ health. Adding to most existing research, this study focuses on the mature sandwich generation – i.e. European individuals aged between 50 to 65 years, who are demographically sandwiched between adult children and older parents. Using data from the Survey of Health, Ageing and Retirement in Europe and fixed effects models we assess the effect of becoming socially sandwiched (a transition and not a status) on respondents’ mental health in according to their status on the labor market. Euro-D and CAS12 are utilized as proxies of individuals psychological wellbeing. It is shown that the effect of becoming socially sandwiched varies by in paid status and gender. The results demonstrate that a transition into social sandwiching has a negative effect on the mental health of working women, while the effect on women who are not in paid work is not statistically significant. The mental health of becoming sandwiched for men who are not in paid work for reasons other than being retired is also negatively affected by becoming socially sandwiched. Whereas, for men who are neither in paid work nor retired, the effect of transitioning into social sandwiching on their mental health is not statistically significant.

